# Celastrol inhibits the proliferation and angiogenesis of high glucose-induced human retinal endothelial cells

**DOI:** 10.1186/s12938-021-00904-5

**Published:** 2021-06-30

**Authors:** Jian Fang, Xiaoke Chang

**Affiliations:** 1 Department of Ophthalmology, Xinchang County People’s Hospital, Shaoxing, 312500 Zhejiang China; 2 Hankou Aier Eye Hospital, No.328, Machang Road, Jianghan District, Wuhan, 430000 Hubei China

**Keywords:** Celastrol, High glucose, Human retinal endothelial cell, HIF1α/VEGF signaling pathway

## Abstract

**Background:**

Diabetic retinopathy (DR) is one of the most common microvascular complications of diabetes. Celastrol plays a certain role in the improvement of various diabetes complications. Therefore, this study aimed to explore whether celastrol inhibited the proliferation and angiogenesis of high glucose (HG)-induced human retinal endothelial cells (hRECs) by down-regulating the HIF1/VEGF signaling pathway.

**Methods:**

The viability and proliferation of hRECs treated with glucose, celastrol or dimethyloxallyl glycine (DMOG) were analyzed by MTT assay. The invasion and tube formation ability of hRECs treated with glucose, celastrol or DMOG were in turn detected by transwell assay and tube formation assay. The expression of HIF1α and VEGF in hRECs after indicated treatment was analyzed by Western blot analysis and RT-qPCR analysis and ICAM-1 expression in hRECs after indicated treatment was detected by immunofluorescence assay

**Results:**

HG induction promoted the proliferation, invasion and tube formation ability and increased the expression of HIF-1α and VEGF of hRECs, which were gradually suppressed by celastrol changing from 0.5 to 2.0 μM. DMOG was regarded as a HIF1α agonist, which attenuated the effect of celastrol on HG-induced hRECs.

**Conclusion:**

Celastrol inhibited the proliferation and angiogenesis of HG-induced hRECs by down-regulating the HIF1α/VEGF signaling pathway.

## Background

Celastrol, mainly derived from the root of *Tripterygium* *wilfordii*, is a quinone methyl triterpene compound, which is one part of *Tripterygium* *wilfordii* [1]. In recent years, researchers have carried out many studies on celastrol at the animal level and cellular level, respectively. It has been reported that celastrol plays an anti-inflammatory role in numerous animal pathological models, such as rheumatoid arthritis, collagen-induced arthritis, Alzheimer’s disease, asthma and systemic lupus erythematosus [2–6]. Especially, a recent discovery that celastrol can prevent and treat insulin resistance and obesity has aroused intense attention. The multi-target action of traditional Chinese medicine has the characteristics of multi-effect and multi-use of one drug, which helps to reduce the risk of multi-drug interaction and adverse drug effects. Celastrol not only reduced blood creatinine and urea nitrogen levels in diabetic rats, but also reduced urinary protein excretion, improved renal pathological damage, and down-regulated p38MAPK and NF-κBp65 expression in diabetic rats [7]. Celastrol could prevent high glucose (HG)-induced podiatric cell damage, inflammation and insulin resistance by restoring HO-1-mediated autophagy, suggesting a therapeutic strategy for diabetic nephropathy (DN) [8]. Celastrol delayed the progression of diabetic liver disease in type 2 diabetic rats by inhibiting the TLR4/MyD88/NF-κB signaling cascade pathway and its downstream inflammatory factors [9]. The expressions of AMPK, PGC1α, Sirt3 and MnSOD in skeletal muscle of diabetic patients were decreased, and celastrol partially regulated the AMPK-PGC1α-Sirt3 signaling pathway to exert antioxidant effect on skeletal muscle [10]. Celastrol plays a certain role in the improvement of various diabetic complications, but its function in diabetic retinopathy (DR) has not been explored.

Diabetes mellitus (DM), being a chronic metabolic disorder, has posed major threats to public health due to its related complications [11]. DR is one of the most common microvascular complications of diabetes and may even lead to vision loss [12]. The general incidence of DR is about one-third of DM patients. The study shows that the number of DR patients will increase from 127 million in 2010 to 191 million in 2030 [13]. Retinal neovascularization has been identified as a key risk factor for severe visual deterioration in DR patients [14]. Therefore, the control of HG-induced neovascularization is the key to preventing the development of DR. Celastrol might inhibit the growth and migration of colorectal cancer cells by inhibiting NOS activity and the angiogenic pathway [15]. Moreover, celastrol significantly down-regulated the expression of lipopolysaccharide (LPS)-induced Toll-like receptor 4 (TLR4) and inhibited the secretion of LPS-induced vascular endothelial growth factor (VEGF) in LP-1 cells. Celastrol inhibited LPS-induced angiogenesis by suppressing TLR4-triggered NF-κB activation [16]. Likewise, through inhibiting CXCR4 expression in HIF-1α-mediated fibroblast-like synoviocyte, inhibitory effects were also exerted by celastrol on hypoxia-induced migration and invasion [17]. Retinal neovascularization is a common cause of vision loss in proliferative diabetic retinopathy, retinopathy of prematurity, and age-related macular degeneration. Samul-tang significantly inhibited retinal neovascularization by down-regulating HIF1, SDF-1, CXCR4 and VEGF [18]. We speculated that celastrol may affect the angiogenesis in DR.

**Fig. 1:**
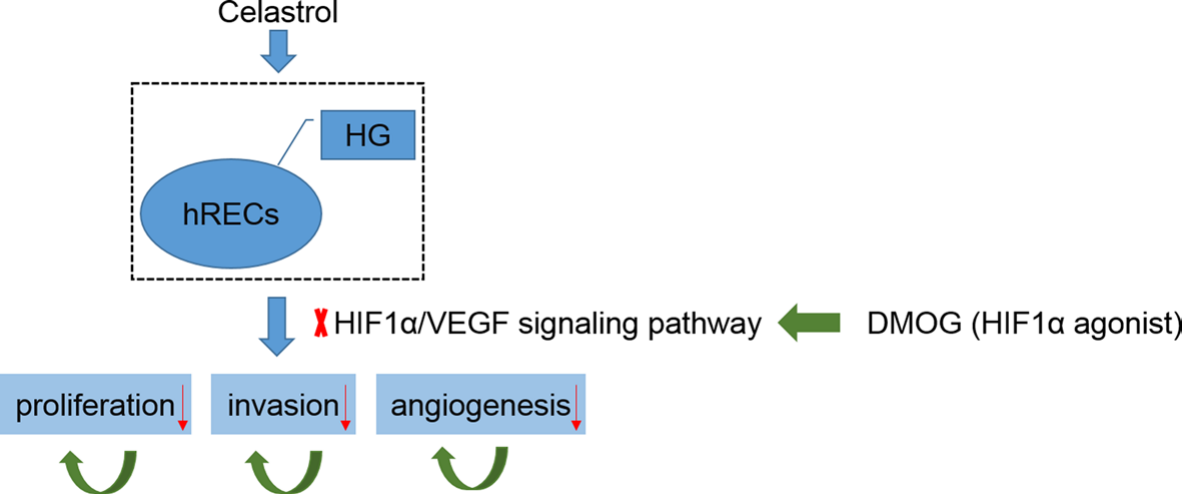
The mechanism of celastrol regulating proliferation, invasion and angiogenesis of HG-induced hRECs

In summary, this study was aimed at exploring the role of celastrol in DR in vitro model of HG-induced human retinal endothelial cells (hRECs) by regulating the proliferation, invasion and angiogenesis through the HIF1α/VEGF signaling pathway. The main content of this study is shown as Fig. 1.

## Results

### Celastrol exerts no effect on the viability of hRECs

**Fig. 2:**
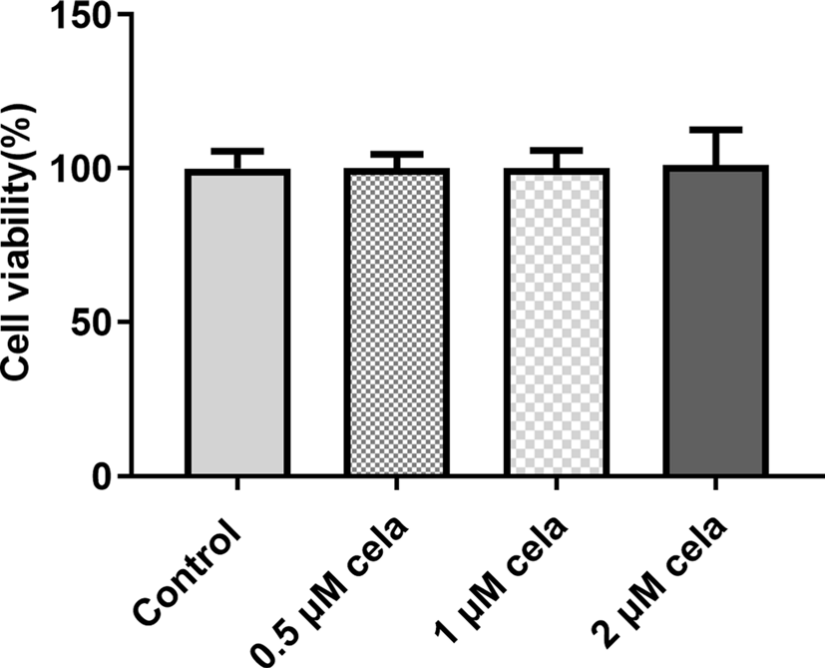
Effect of celastrol on the viability of hRECs. The viability of hRECs treated with different concentrations of celastrol was detected by MTT assay. Cela: celastrol

hRECs were treated with celastrol at different concentrations (0.5 μM, 1.0 μM and 2.0 μM) for 24 h. No obvious differences were observed among these four groups, which indicated that celastrol did not affect the viability of hRECs (Fig. 2).

### Celastrol inhibits the proliferation and invasion of HG-induced hRECs

**Fig. 3:**
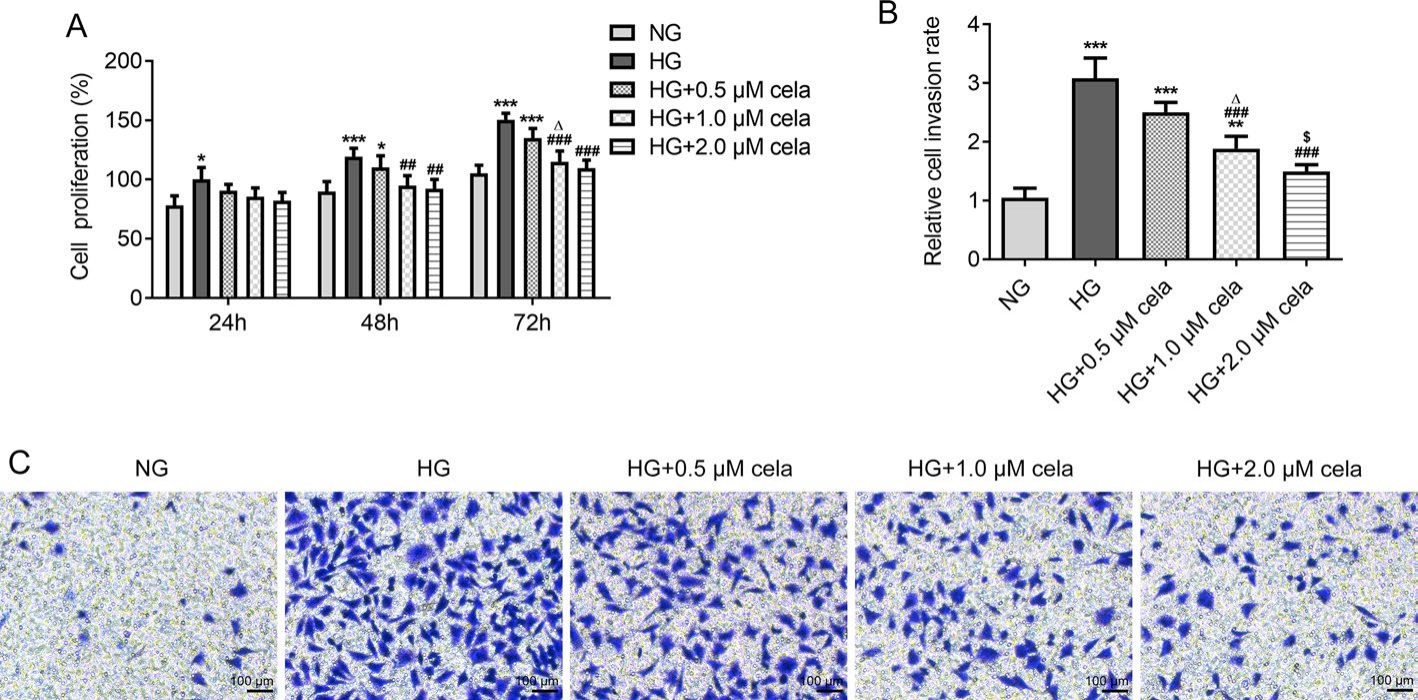
Celastrol inhibits the proliferation and invasion of HG-induced hRECs. **A** The proliferation of hRECs induced with HG and treated with different concentrations of celastrol was detected by MTT assay. **B** The invasion of hRECs induced with HG and treated with different concentrations of celastrol was observed by Transwell assay. **C** Optical micrographs of invasion of hRECs induced with HG and treated with different concentrations of celastrol. *P < 0.05, **P < 0.01 and ***P < 0.001 vs. NG group. ^*##*^*P* < 0.01 and ^*###*^*P* < 0.001 vs. HG group. ^*∆*^*P* < 0.05 vs. HG + 0.5 μM cela group. ^$^*P* < 0.05 vs. HG + 1.0 μM cela group. Cela: celastrol

HG promoted the proliferation of hRECs from 24 to 72 h. With the increasing concentration and time, the proliferation and invasion of HG-induced hRECs were inhibited by celastrol (Fig. 3A). The invasion of hRECs was also promoted by HG while celastrol at 1.0 μM and 2.0 μM could significantly suppressed the invasion of hRECs (Fig. 3B, C).

### Celastrol inhibits the angiogenesis of HG-induced hRECs

**Fig. 4:**
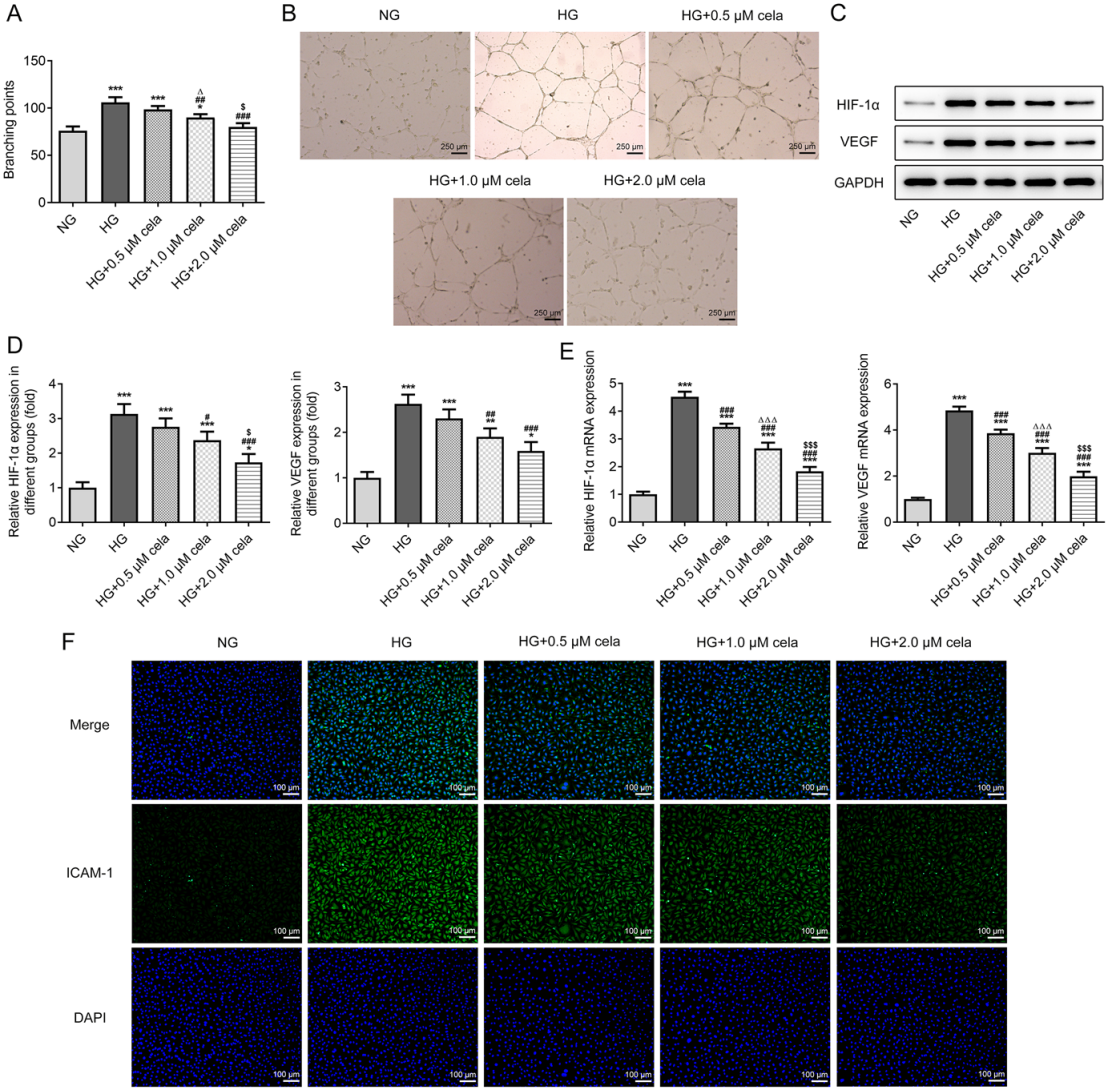
Celastrol inhibits the angiogenesis of HG-induced hRECs. **A** The branching points in hRECs induced with HG and treated with different concentrations of celastrol were observed by tube formation assay. **B** Inverted micrographs of branching points in hRECs induced with HG and treated with different concentrations of celastrol. **C** Representative western blots and **D** quantitative analysis for expression of HIF-1α and VEGF in hRECs induced with HG and treated with different concentrations of celastrol was detected by Western blot analysis. **E** The mRNA expression of HIF-1α and VEGF in hRECs induced with HG and treated with different concentrations of celastrol was detected by RT-qPCR analysis. **F** The expression of ICAM-1 in hRECs induced with HG and treated with different concentrations of celastrol was detected by immunofluorescence assay. *P < 0.05, **P < 0.01 and ***P < 0.001 vs. NG group. ^#^*P* < 0.05, ^##^*P* < 0.01 and ^###^*P* < 0.001 vs. HG group. ^∆^*P* < 0.05 vs. HG + 0.5 μM cela group. ^$^*P* < 0.05 and ^$$$^*P* < 0.001 vs. HG + 1.0 μM cela group. Cela: celastrol

The branching points in hRECs were increased after HG induction, while were later reduced by celastrol (Fig. 4A, B). The protein and mRNA expression of HIF-1α and VEGF were increased in hRECs induced by HG, which were gradually reversed by increasing concentration of celastrol (Fig. 4C–E). Figure 4F indicates that ICAM-1 expression was increased in HG-induced hRECs and ICAM-1 expression was gradually decreased with the increasing concentration of celastrol. 2.0 μM celastrol was selected for the subsequent experiment.

### Celastrol inhibits the proliferation and invasion of HG-induced hRECs by down-regulating the HIF1α/VEGF signaling pathway

**Fig. 5:**
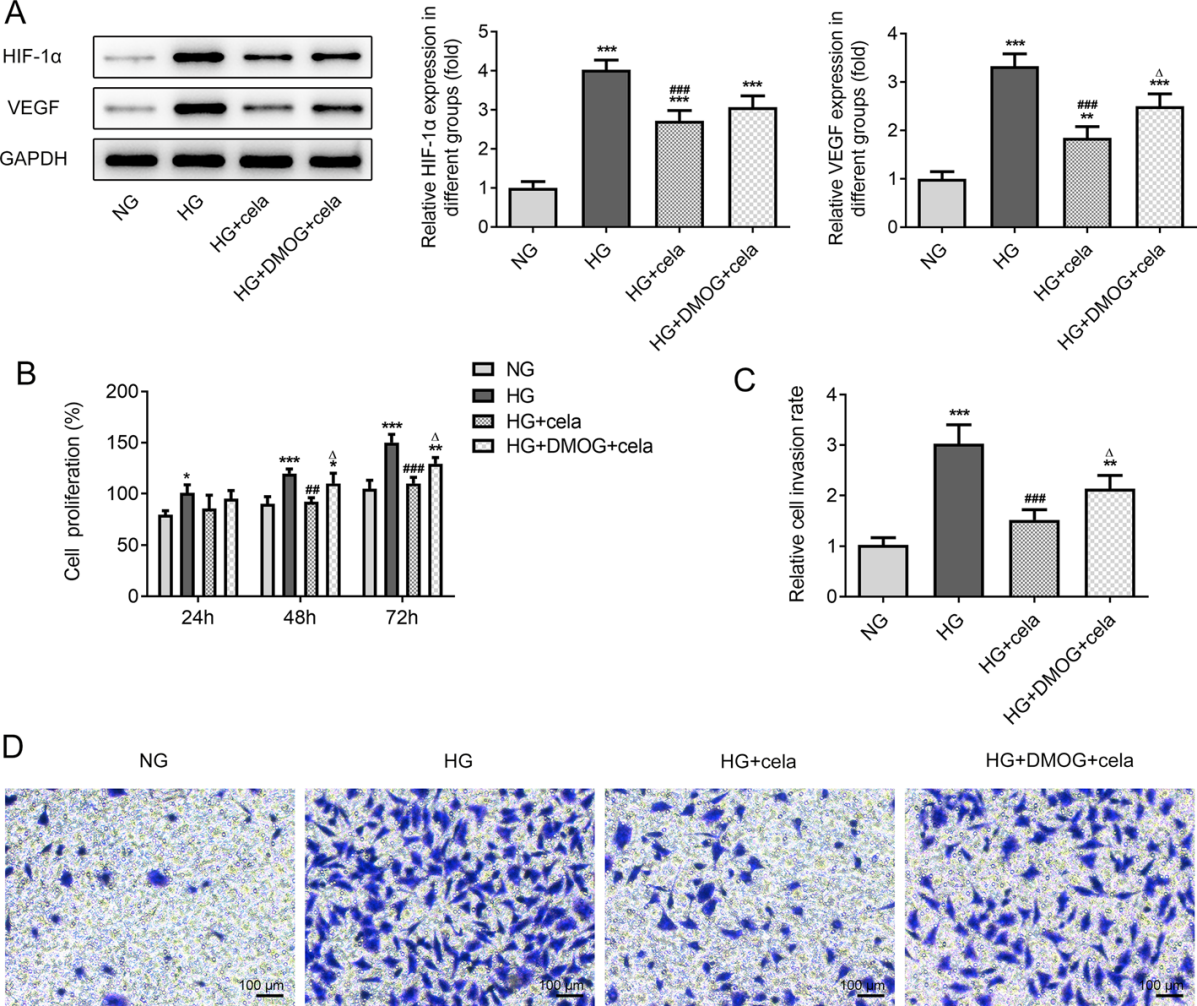
Celastrol inhibits the proliferation and invasion of HG-induced hRECs by down-regulating the HIF1α/VEGF signaling pathway. **A** The protein expression of HIF-1α and VEGF in HG-induced hRECs treated with DMOG and celastrol was detected by Western blot analysis. **B** The proliferation of HG-induced hRECs treated with DMOG and celastrol was observed by MTT assay. **C** The invasion of HG-induced hRECs treated with DMOG and celastrol was observed by Transwell assay. **D** Optical micrographs of invasion of HG-induced hRECs treated with DMOG and celastrol. *P < 0.05, **P < 0.01 and ***P < 0.001 vs. NG group. ^##^*P* < 0.01 and ^###^*P* < 0.001 vs. HG group. ^∆^*P* < 0.05 and ^∆∆∆^*P* < 0.001 vs. HG + cela group. Cela: celastrol

DMOG improved the protein expression of HIF1α and VEGF in HG-induced hRECs treated with celastrol (Fig. 5A). DMOG increased the proliferation (Fig. 5B) and invasion (Fig. 5C, D) of HG-induced hRECs treated with celastrol.

### Celastrol inhibits the angiogenesis of HG-induced hRECs by down-regulating the HIF1α/VEGF signaling pathway

**Fig. 6:**
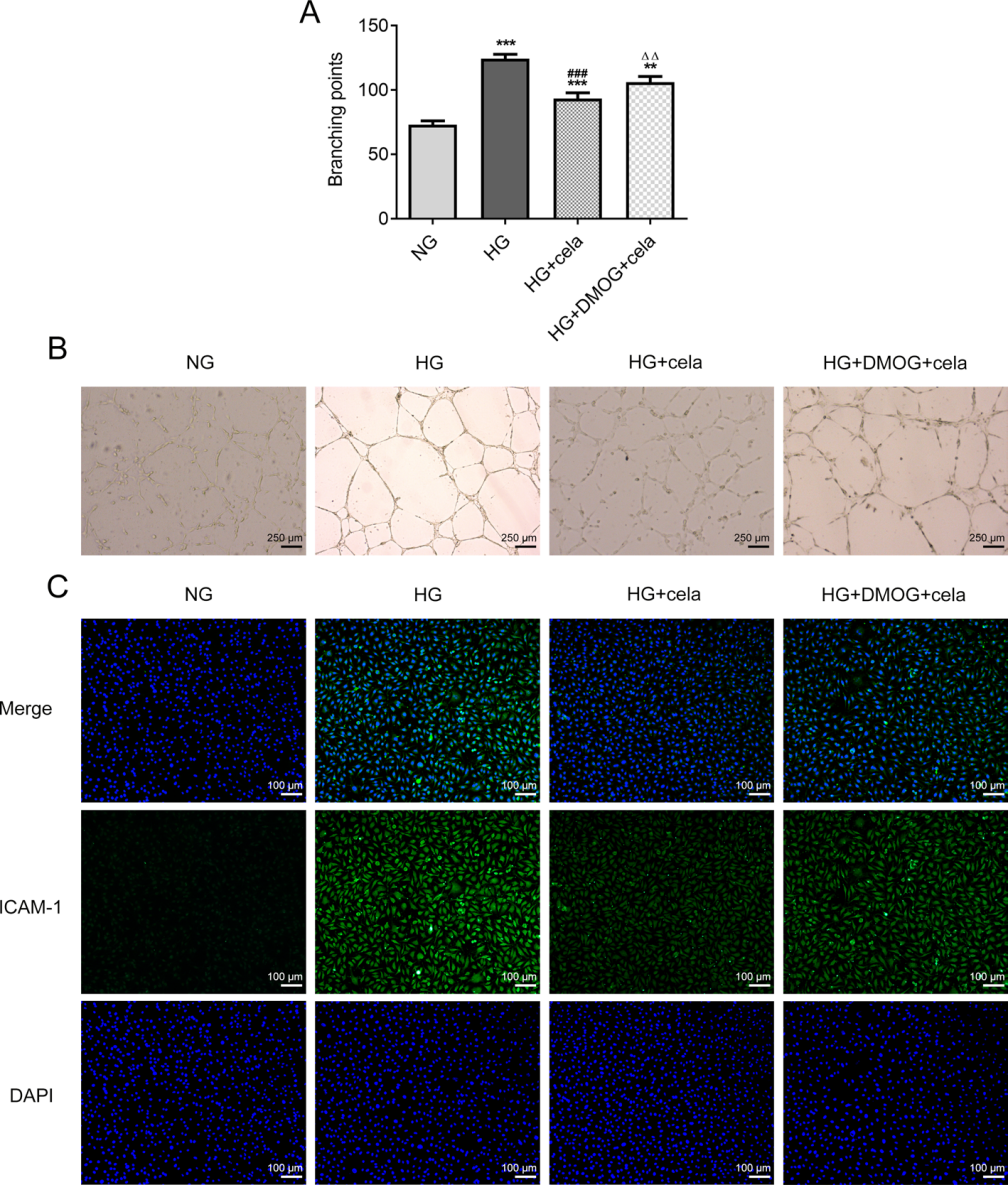
Celastrol inhibits the angiogenesis of HG-induced hRECs by down-regulating the HIF1α/VEGF signaling pathway. **A** The branching points in HG-induced hRECs treated with DMOG and celastrol were observed by tube formation assay. **B** Inverted micrographs of branching points in HG-induced hRECs treated with DMOG and celastrol. **C** The expression of ICAM-1 in HG-induced hRECs treated with DMOG and celastrol was detected by immunofluorescence assay. **P < 0.01 and ***P < 0.001 vs. NG group. ^###^*P* < 0.001 vs. HG group. ^∆∆^*P* < 0.01 vs. HG + cela group. Cela: celastrol

DMOG increased the branching points in HG-induced hRECs treated with celastrol (Fig. 6A, B). DMOG improved the expression of ICAM-1 in HG-induced hRECs treated with celastrol (Fig. 6C).

## Discussion

DR, as a common microvascular complication in diabetic patients, is the main factor that leads to blindness and seriously threatens the life quality of patients [19]. Study showed that retinal angiogenesis was an important mechanism of the pathogenesis of DR [20].

Studies showed that celastrol could not only reduce blood glucose [21] and improve insulin resistance [22], but also have effects of prevention and treatment on the complications of diabetes. Celastrol reduced levels of Aβ protein deposition and GFAP in the hippocampus of diabetic rats, thereby improving the cognitive impairment caused by diabetes [23]. The above experimental studies showed that celastrol had the function of improving the complications of diabetes. In addition, angiogenesis and angiogenesis simulation were considered to be the main processes that ensure the blood supply of tumors during the growth and metastasis of glioblastoma. Furthermore, celastrol reduced the angiogenesis-related proteins CD31, vascular endothelial growth factor receptor (VEGFR) 2, angiogenin (Ang) 2 and VEGFA [24]. In this study, celastrol inhibited the proliferation, invasion and angiogenesis of HG-induced hRECs, which might indicate that celastrol could alleviate the diabetic retinopathy, but further study should be constructed in vivo experiment.

High glucose environment caused by diabetes will lead to insufficient blood supply to the retina, which will consequently be in a state of relative hypoxia [25]. As an important oxygen content receptor and regulatory protein in the body, HIF-1α plays an important role in the regulation of angiogenesis, extracellular matrix metabolism, inflammation and other processes [26]. VEGF is the most important regulator of the formation of new blood vessels. In addition, VEGF is an important downstream target gene of the HIF-1α signaling pathway [27]. It can also increase the permeability of blood vessels, induce the proliferation and migration of vascular endothelial cells, and generate extracellular matrix, thus promoting the formation of new blood vessels [28]. HIF-1α upregulates VEGF through direct transcription and plays a leading role in neovascularization. In HIF-1α knocked out mice model, production of VEGF and ICAM-1 was reduced and vascular leakage and neovascularization were also significantly decreased in the HIF-1α knocked out mice compared with wild-type mice [29]. Zhang et al. [30] interfered HIF-1α with small molecular RNA in mice, and found that silencing of HIF-1α could reduce VEGF expression and inhibit angiogenesis. In chemical (CoCl_2_) hypoxia-induced hRECs, the expression of HIF-1α and VEGF was increased and activation of HIF-1α could upregulate the expression of VEGF [31]. HG-induced increased expression of VEGF, leading to the induction of apoptosis of hRECs via binding to VEGFR2 [32]. In this study, celastrol decreased the expression of HIF-1α and VEGF in HG-induced hRECs and increased expression of HIF-1α. Moreover, VEGF promoted by DMOG increased the branching points to promote the proliferation and angiogenesis of HG-induced hRECs.

## Conclusion

The present study indicated that celastrol inhibited the proliferation and angiogenesis of HG-induced hRECs by down-regulating the HIF1α/VEGF signaling pathway. Moreover, DMOG, as a HIF1α agonist, could weaken the inhibition effect of celastrol on the proliferation and angiogenesis of HG-induced hRECs. This new finding could provide a theoretical basis for the development of DR drugs. However, the present study is on the basis of cell experiment and the underlying mechanisms by which celastrol improves DR requires additional animal experiment and clinical investigation in future.

## Materials and methods

### Cell culture and treatment

Human retinal endothelial cells (hRECs) were purchased from Xuanke biological technology Co., Ltd. (Shanghai, China) and cultured in Endothelial Basal Medium -2 (EBM-2) medium containing 10% fetal bovine serum (FBS; Gibco; Thermo Fisher Scientific, Inc.) at 37 °C with 5% CO_2_. For normal glucose (NG) condition, the concentration of glucose in EBM-2 medium was 5 mM. For HG condition, the concentration of glucose in complete medium was 25 mM. The cells were kept in these conditioned medium for 48 h. Celastrol (99% purity) was purchased from Pharmacodia Co., Ltd. (Beijing, China) and hRECs were treated with celastrol with a gradient of 0.5 μM, 1.0 μM and 2.0 μM.

### MTT assay

When the confluence of hRECs reached 90%, cells were inoculated onto 96-well plates, and the number of cells in each well was 3 × 10^3^. After inoculation, the cells were cultured in EBM-2 medium at 37 °C with 5% CO_2_. After the cell adhered to the wall, the different concentrations of celastrol were added to the normal cells followed by a 24-h incubation.

Another experiment was that hRECs were cultured in EBM-2 medium with 5 mM glucose (NG) or 25 mM glucose (HG) at 37 °C with 5% CO_2_ after inoculation. After the cells adhered to the wall, the different concentrations of celastrol or dimethyloxallyl glycine (DMOG, 1 mmol/L) were added to the HG-induced cells which were incubated for 24 h. DMOG was regarded as a HIF1α agonist [33].

hRECs of each group were added with 50 μL 0.5 mg/mL MTT solution (Beyotime) at a specific time. Then, these cells were incubated at 37 °C with 5% CO_2_ for 4 h. After that, the supernatant was removed and 200 μL dimethyl sulfoxide (DMSO) was added to dissolve the purple crystal formed by the cells. The optical density (OD) value at 490 nm was determined by a microplate reader Elx808 (BioTek Instruments, Inc.).

### Transwell assay

After indicated treatment, 1 × 10^6^ cells/mL cell suspension was prepared with serum-free medium. Then, the cell suspension was added to the upper chamber of 24-well transwell inserts (8 μm pore size, Costar) covered with artificial basement membrane. As for the lower chamber, the medium containing 20% FBS was added, and the follow-up culture was performed at 37 °C for 24 h. The cells at the bottom of the upper chamber were stained with 0.5% crystal violet and the cells inside the upper chamber were removed with a cotton swab. The cells were observed and counted under a CKX31 inverted light microscope (Olympus Corporation).

### hRECs’ tube formation assay

The Matrigel™ Matrix glue was slowly injected into a 96-well plate placed in an ice bath with a pre-cooled nozzle. The volume of injection in each well was 50 μL. The Matrigel™ Matrix glue was then placed in an incubator for 1 h to make Matrigel and Matrix polymerized. Then, the cultured cells were inoculated into a 96-well plate at a density of 3 × 10^3^ cells per well. After inoculation, these cells were added with different medium and drugs and then continuously cultured for another 6 h. The tubular structure was recorded by a CKX31 inverted light microscope (Olympus Corporation) and the branching points were visualized by ImageJ software (National Institutes of Health).

### Reverse transcription-quantitative PCR (RT-qPCR) analysis

After indicated treatment, total RNA in cells at logarithmic growth was extracted with Trizol reagent. After the purity and concentration of RNA were detected, the RNA was reversely transcribed into cDNA using the PrimeScript™ RT Master Mix (Takara). qPCR was subsequently performed according to the instructions of the One Step TB Green^®^ PrimeScript™ RT-PCR Kit (Takara). The following primer sequences were used for the qPCR: HIF-1α forward, 5'-TGAAGTGTACCCTACCCTAACTAGCCG-3' and reverse, 5'-AATCAGCACCAAGCAGGTCATAG-3'; VEGF forward, 5'-GTCACTATG CAGATCATGCGGA-3' and reverse, 5'-GTCACTATGCAGATCATGCGGA-3'; GAPDH forward, 5'-AAGAGGGATGCTGCCCTTAC-3' and reverse, 5'-TACGGCCAAATCCGTTCACA-3'. The expression of HIF-1α and VEGF was analyzed by 2^−∆∆Ct^ method normalized to endogenous control of GAPDH [34].

### Western blot analysis

After indicated treatment, cells were rinsed with phosphate buffered saline (PBS), and then cell lysates containing protease inhibitors were added to cells to extract the total proteins, which were degenerated at 100 °C for 5 min. Equivalent proteins were separated by sodium dodecyl sulfate–polyacrylamide gel electrophoresis (SDS-PAGE). After separated proteins were obtained, they were transferred to polyvinylidene fluoride (PVDF) membrane. Prior to the addition of primary antibodies, membranes were blocked by 5% bovine serum albumin (BSA) for 1 h. The corresponding primary antibodies utilized herein were as follows: anti-HIF-1α (ab179483; dilution, 1:1000; Abcam), anti-VEGF (Cat. No. OPA1-10110; dilution, 1:200; Thermo Fisher Scientific, Inc.) and anti-GAPDH (ab9485; dilution, 1:2500; Abcam). On the next day following the overnight primary antibody incubation, the detection of target protein is conducted with the secondary antibody (horseradish peroxidase-conjugated IgG) (ab6721; dilution, 1:2,000; Abcam). Subsequently, membranes were incubated at room temperature for 1.5 h. Finally, the chemiluminescent solution (Bio-Rad Laboratories, Inc.) was added to the gel imager for exposure and photography, and the gray value was counted and the relative expression of the proteins was detected.

### Immunofluorescence assay

After indicated treatment, the cell slides were fixed with 4% paraformaldehyde, treated with 0.5% tritonx-100 for 10 min and 2% H_2_O_2_ for 20 min. These cells were sealed for 30 min at room temperature, depending on goat serum. Then, ICAM-1 primary antibody (ab214944; dilution, 1:100; Abcam) diluted with 0.01 mol/L PBS was added to the cell slides which were incubated overnight at 4 °C. After washing, secondary immunofluorescence was performed using FITC-tagged secondary antibody, and the incubation lasted for 2 h with the temperature controlled at 37 °C. Finally, DAPI (Keygen Biotech) was added at a final concentration of 5 mg/L to stain the slides in the dark and then cell slides were sealed, visualized and photographed under a Nikon A1 confocal laser scanning microscope (Nikon).

### Statistical analysis

The continuous variables in this study were expressed as mean ± standard deviation (SD). One-way analysis of variance (ANOVA) followed by Tukey’s post hoc test was used for comparison between multiple groups. SPSS software was used for data analysis (version 20.0; SPSS Inc., USA). *P* < 0.05, *P* < 0.01 and *P* < 0.001 were all considered to be statistically significant.

## Data Availability

The experimental data will be available on the request.
